# A multicenter, prospective validation study of the Japanese Association for Acute Medicine disseminated intravascular coagulation scoring system in patients with severe sepsis

**DOI:** 10.1186/cc12783

**Published:** 2013-06-20

**Authors:** Satoshi Gando, Daizoh Saitoh, Hiroshi Ogura, Seitaro Fujishima, Toshihiko Mayumi, Tsunetoshi Araki, Hiroto Ikeda, Joji Kotani, Shigeki Kushimoto, Yasuo Miki, Shin-ichiro Shiraishi, Koichiro Suzuki, Yasushi Suzuki, Naoshi Takeyama, Kiyotsugu Takuma, Ryosuke Tsuruta, Yoshihiro Yamaguchi, Norio Yamashita, Naoki Aikawa

**Affiliations:** 1 Division of Acute and Critical Care Medicine, Department of Anesthesiology and Critical Care Medicine, Hokkaido University Graduate School of Medicine, N15W7, Kita-ku, Sapporo, Japan; 2 Division of Traumatology, Research Institute, National Defense Medical College, Japan; 3 Department of Traumatology and Acute Critical Care Medicine, Osaka University Medical School, Japan; 4 Department of Emergency & Critical Care Medicine, School of Medicine, Keio University, Japan; 5 Emergency Center, Department of Emergency and Critical Care Medicine, Ichinomiya Municipal Hospital, Japan; 6 Department of Emergency &Critical Care Medicine, Trauma Center St. Mary's Hospital, Japan; 7 Department of Emergency Medicine, Trauma and Resuscitation Center, Teikyo University School of Medicine, Japan; 8 Department of Emergency, Critical Care and Disaster Medicine, Hyogo College of Medicine, Japan; 9 Division of Emergency Medicine, Tohoku University Graduate School of Medicine, Japan; 10 Advanced Critical Care Center, Aichi Medical University Hospital, Japan; 11 Department of Emergency and Critical Care Medicine, Nippon Medical School, Japan; 12 Department of Acute Medicine, Kawasaki Medical School, Japan; 13 Department of Critical Care Medicine, Iwate Medical University, Japan; 14 Department of Emergency and Acute Intensive Care Medicine, Fujita Health University, Japan; 15 Emergency and Critical Center, Kawasaki Municipal Hospital, Japan; 16 Advanced Medical Emergency & Critical Care Center, Yamaguchi University Hospital, Japan; 17 Department of Trauma and Critical Care Medicine, Kyorin University, School of Medicine, Japan; 18 Department of Emergency & Critical Care Medicine, School of Medicine, Kurume University, Japan

## Abstract

**Introduction:**

To validate the Japanese Association for Acute Medicine (JAAM) disseminated intravascular coagulation (DIC) scoring system in patients with severe sepsis, we conducted a multicenter, prospective study at 15 critical care centers in tertiary care hospitals.

**Methods:**

This study included 624 severe sepsis patients. JAAM DIC was scored on the day of diagnosis of severe sepsis (day 1) and day 4. Scores for disease severity and organ dysfunction were also evaluated.

**Results:**

The prevalence of JAAM DIC was 46.8% (292/624), and 21% of the DIC patients were scored according to the reduction rate of platelets. The JAAM DIC patients were more seriously ill and exhibited more severe systemic inflammation, a higher prevalence of multiple organ dysfunction syndrome (MODS) and worse outcomes than the non-DIC patients. Disease severity, systemic inflammation, MODS and the mortality rate worsened in accordance with an increased JAAM DIC score on day 1. The Kaplan-Meier curves demonstrated lower 1-year survival in the JAAM DIC patients than in those without DIC (log-rank test *P *<0.001). The JAAM DIC score on day 1 (odds ratio = 1.282, *P *<0.001) and the Delta JAAM DIC score (odds ratio = 0.770, *P *<0.001) were independent predictors of 28-day death. Dynamic changes in the JAAM DIC score from days 1 to 4 also affected prognoses. The JAAM DIC scoring system included all patients who met the International Society on Thrombosis and Haemostasis overt DIC criteria on day 1. The International Society on Thrombosis and Haemostasis scoring system missed a large number of nonsurvivors recognized by the JAAM scoring system.

**Conclusions:**

The JAAM DIC scoring system exhibits good prognostic value in predicting MODS and poor prognosis in patients with severe sepsis and can detect more patients requiring treatment. Conducting repeated daily JAAM scoring increases the ability to predict the patient's prognosis.

## Introduction

Disseminated intravascular coagulation (DIC) is a frequent complication of systemic inflammatory response syndrome (SIRS) [1]. Sepsis, defined as infection-induced SIRS, almost invariably leads to hemostatic abnormalities ranging from insignificant coagulopathy to severe DIC [2]. DIC results in the widespread formation of fibrin thrombosis, microvascular occlusion and reduced oxygen delivery to cells and tissues, leading to multiple organ dysfunction syndrome (MODS) [3]. A prospective epidemiologic study clearly demonstrated that a clinical progression from SIRS to severe sepsis and septic shock increases the prevalence of DIC, organ dysfunction and the risk of death [4]. DIC associated with sepsis is therefore a syndrome that should be diagnosed and treated early [5,6].

Scoring systems for DIC developed based on the Japanese Ministry of Health and Welfare scoring system have been independently proposed by the Japanese Association for Acute Medicine (JAAM) and the International Society on Thrombosis and Haemostasis (ISTH). These scoring systems have been prospectively validated in diverse patient populations [7-11]. Three subgroup analyses of large randomized controlled phase 3 studies evaluating the treatment effects of anticoagulant drugs in patients with severe sepsis used the JAAM and ISTH DIC scoring systems for the diagnosis of DIC [12-14]. Both the JAAM and ISTH scoring systems were effectively used to diagnose DIC and select proper patient groups who require DIC treatment, although some modifications were made to the latter system. Three recently published guidelines for the diagnosis and management of DIC variably recommend the JAAM and ISTH DIC scoring systems based on literature reviews and analyses [15-17].

DIC is not a disease in itself, but rather a syndrome that always develops secondary to various underlying disorders. DIC scoring systems are therefore usually validated in heterogeneous groups of patients, which may result in different evaluations of DIC diagnostic properties and recommendations in the scoring systems. In the present study, the JAAM Sepsis Registry Study Group prospectively validated the JAAM DIC scoring system in patients with severe sepsis, the leading cause of DIC, in a critical care setting.

## Materials and methods

This study was prospectively conducted by the JAAM Sepsis Registry Study Group as part of a multicenter prospective survey of severe sepsis in Japan [18]. Both the JAAM and the Ethics Committees of the participating hospitals approved the study protocol. The data collection was performed as a routine clinical workup without any interventions, and the data management and statistical analyses were processed anonymously. For these reasons, both the JAAM and the Ethics Committees of each hospital waived written informed consent.

### Patients

The patients recruited for this prospective validation study were registered at 15 critical care centers in tertiary care hospitals during a 1-year period from 1 June 2010 to 31 May 2011. All patients admitted to the ICU without any exclusion criteria were enrolled when they were diagnosed as having severe sepsis.

### Definitions

SIRS, sepsis, severe sepsis and septic shock were defined according to the American College of Chest Physicians/Society of Critical Care Medicine consensus conference and its revised version published in 2003 [19,20]. The disease severity of the patients was evaluated according to the Acute Physiology and Chronic Health Evaluation (APACHE) II score at the time of enrollment [21]. Organ dysfunction was assessed according to the Sequential Organ Failure Assessment (SOFA) score [22]. MODS was defined as a SOFA score ≥12 [22]. A DIC diagnosis was made on the basis of the JAAM DIC diagnostic criteria (Table 1) [8,10]. The Delta JAAM DIC score was calculated using the day 1 JAAM DIC score minus the day 4 JAAM DIC score. Overt DIC scores based on the ISTH scoring system were also calculated (Table 2) [9]. ISTH overt DIC was defined as JAAM DIC patients who simultaneously met the ISTH overt DIC criteria on day 1 in the present study. The fibrin/fibrinogen degradation product (FDP) was used as the fibrin-related marker for the ISTH criteria. No increase, moderate increase and strong increase were defined as FDP <10, 10 ≤ FDP <25, and FDP ≥25 mg/l, respectively. When the total score was ≥4 and ≥5, a diagnosis was established using the JAAM and ISTH criteria, respectively. The outcome measures were the 28-day and hospital all-cause mortality rates.

**Table 1:** Scoring system for disseminated intravascular coagulation by the Japanese Association for Acute Medicine

	Score
SIRS criteria	
≥3	1
0 to 2	0
Platelet counts	
<80 × 10^9^/l or >50% decrease within 24 hours	3
≥80 <120 × 10^9^/l or >30% decrease within 24 hours	1
≥120 × 10^9^/l	0
Prothrombin time (value of patient/normal value)	
≥1.2	1
<1.2	0
Fibrin/fibrinogen degradation products	
≥25 mg/l	3
≥10 <25 mg/l	1
<10 mg/l	0
Diagnosis	
Disseminated intravascular coagulation	≥4

SIRS, systemic inflammatory response syndrome.

**Table 2:** Scoring system for overt disseminated intravascular coagulation proposed by International Society on Thrombosis and Haemostasis

	Score
Platelet counts	
<50 × 10^9^/l	2
≥50 <100 × 10^9^/l	1
≥100 × 10^9^/l	0
Elevated fibrin-related marker^a^	
Strong increase	3
Moderate increase	2
No increase	0
Prolonged prothrombin time	
≥6 seconds	2
≥3 <6 seconds	1
<3 seconds	0
Fibrinogen level	
<100 g/ml	1
≥100 g/ml	0
Calculate score	
If >5, compatible with overt DIC; repeat scoring daily	
If <5, suggestive (not affirmative) for nonovert DIC; repeat next 1 to 2 days.	

DIC, disseminated intravascular coagulation. ^a^For example, soluble fibrin monomers/fibrin degradation products.

### Data sampling

Prospective blood sampling was performed on admission to the ICU and daily thereafter as part of a routine clinical and laboratory workup using established standard laboratory techniques. APACHE II, SOFA, SIRS and both the JAAM and ISTH DIC scores were assessed, and the platelet counts and coagulation variables necessary to diagnose DIC were collected on the day of enrollment (day 1). Evaluation of the SOFA, SIRS and both the JAAM and ISTH DIC scores on day 4 was also recorded.

### Statistical analysis

All measurements are expressed as the mean ± standard deviation. The IBM SPSS 20.0 for MAC OSX software program (IBM Japan, Tokyo, Japan) was used for the statistical analyses and calculations. Comparisons between two groups were made with the unpaired Student's *t *test for parametric data or Mann-Whitney's U-test for nonparametric data, and either the chi-square test or Fisher's exact test was used when required. To compare multiple groups, a factorial analysis of variance with the *post-hoc *Dunnett's *t *test and multiple chi-square tests were applied. The relationships between dependent and independent variables were analyzed using a stepwise logistic regression analysis with backward elimination based on the likelihood ratio. The results of the regressions were reported as the odds ratios and 95% confidence intervals. Survival curves were derived according to the Kaplan-Meier method and compared using the log-rank test. Differences with *P *<0.05 were considered statistically significant.

## Results

### Baseline characteristics and outcomes of the patients

During the 1-year registration period, a total of 14,417 patients were admitted to 15 critical care centers, of whom 624 (4.3%) patients were diagnosed as having severe sepsis and were enrolled in the study. Of these 624 patients, 292 (46.8%) were diagnosed with DIC according to the JAAM DIC scoring system. As shown in Table 3, the JAAM DIC patients exhibited a higher prevalence of septic shock and positive blood cultures, in addition to higher SIRS, APACHE II and SOFA scores, which resulted in a greater incidence of MODS and poorer prognoses compared with patients without DIC. We further confirmed that there was a significant difference in the JAAM DIC scores on day 1 between survivors (3.4 ± 2.2) and nonsurvivors (4.3 ± 2.0) amongst all patients (*P *<0.001). The prevalence of JAAM DIC was also different between survivors (201/480, 41.9%) and nonsurvivors (91/144, 63.2%) (*P *<0.001). The survival curves of the two groups derived according to the Kaplan-Meier method demonstrated that the survival rate of the patients with JAAM DIC was significantly lower than that of the patients without DIC (log-rank test *P *<0.001).

**Table 3:** Characteristics of JAAM DIC and non-DIC patients on the day of inclusion (day 1)

	JAAM DIC (*n *= 292)	Non-DIC (*n *= 332)	*P *value
Age (years)	69 ± 18	69 ± 15	0.575
Gender (male/female)	181/111	210/122	0.744
Septic shock (%)	56.2	35.5	<0.001
Positive blood culture (%)	49.5	33.9	<0.001
JAAM DIC score	5.6 ± 1.3	1.9 ± 0.9	<0.001
Platelet counts (× 10^9^/l)	89 ± 88	215 ± 111	<0.001
Prothrombin time (seconds)	19.5 ± 9.5	16.1 ± 5.1	<0.001
Prothrombin time ratio	1.63 ± 0.64	1.40 ± 0.56	<0.001
Fibrinogen (g/l)	3.96 ± 1.96	4.83 ± 1.99	<0.001
FDP (mg/l)	62.5 ± 104.7	10.6 ± 6.7	<0.001
SIRS score	3.3 ± 0.8	3.1 ± 0.9	0.007
APACHE II score	25.2 ± 8.5	21.9 ± 7.9	<0.001
SOFA score	10.6 ± 3.8	6.7 ± 3.3	<0.001
MODS (%)	65.4	40.4	<0.001
28-day outcome (death/%)	91/31.2	53/16.0	<0.001
Hospital outcome (death/%)	112/38.4	72/21.7	<0.001

APACHE, Acute Physiology and Chronic Health Evaluation; DIC, disseminated intravascular coagulation; FDP, fibrin/fibrinogen degradation products; JAAM, Japanese Association for Acute Medicine; MODS, multiple organ dysfunction syndrome; SIRS, Systemic Inflammatory Response Syndrome; SOFA, Sequential Organ Failure Assessment.

### Relationships between DIC scores, severity scores and outcomes

We observed stepwise deterioration in the severity scores associated with significant increases in the prevalence of MODS and the mortality rate in accordance with an increase in the JAAM DIC scores on day 1 from 0 to a maximum of 8 (Table 4). In particular, the SOFA scores on day 1, the maximum SOFA scores observed during the study period and the prevalence of MODS increased in parallel with the increase in the DIC scores.

**Table 4:** Disease severity, organ dysfunction and mortality for JAAM DIC score on inclusion day (day 1)

	JAAM DIC score								
	
	0 (*n *= 19)	1 (*n *= 89)	2 (*n *= 143)	3 (*n *= 81)	4 (*n *= 59)	5 (*n *= 105)	6 (*n *= 59)	7 (*n *= 19)	8 (*n *= 50)
APACHE II score	22.3 ± 7.8	20.2 ± 7.6	21.8 ± 7.5	23.7 ± 8.6	23.4 ± 7.9	26.1 ± 9.1^bc^	25.1 ± 7.0^b^	25.0 ± 9.1	26.0 ± 9.2^b^
SIRS score	1.7 ± 0.5	2.8 ± 1.0	3.2 ± 0.7	3.4 ± 0.7	2.9 ± 1.0^ad^	3.3 ± 0.7^ab^	3.5 ± 0.5^ab^	2.7 ± 1.0^a^	3.6 ± 0.5^abc^
SOFA score	6.5 ± 3.7	5.7 ± 3.3	6.7 ± 2.9	8.0 ± 3.5	9.4 ± 3.4^bc^	10.0 ± 4.2^abcd^	11.4 ± 3.2^abcd^	11.5 ± 3.4^abcd^	11.9 ± 3.6^abcd^
SOFA peak score during study period	6.8 ± 3.8	6.1 ± 3.4	7.4 ± 3.3	8.7 ± 3.6	10.0 ± 3.5^bc^	11.0 ± 4.2^abcd^	11.9 ± 3.3^abcd^	12.3 ± 3.1^abcd^	13.0 ± 3.8^abcd^
MODS (%)	42.1	32.6	37.1	54.3	55.9^bc^	61.9^bc^	72.9^abcd^	63.2^b^	76.0^abcd^
28-day mortality (%)	5.3	10.1	18.9	19.8	27.1^b^	37.1^abcd^	27.1^ab^	31.6^ab^	28.0^ab^
Hospital mortality (%)	10.5	14.6	25.2	25.9	30.5^ab^	43.8^abcd^	35.6^ab^	42.1^ab^	38.0^ab^

APACHE, Acute Physiology and Chronic Health Evaluation; DIC, disseminated intravascular coagulation; JAAM, Japanese Association for Acute Medicine; MODS, multiple organ dysfunction syndrome; SIRS, Systemic Inflammatory Response Syndrome; SOFA, Sequential Organ Failure Assessment. Analyses of variance of APACHE II, SIRS and SOFA are as follows: *P *<0.001, ^a^*P *<0.05 versus 0, ^b^*P *<0.05 versus 1, ^c^*P *<0.05 versus 2, ^d^*P *<0.05 versus 3.

**Figure 1:**
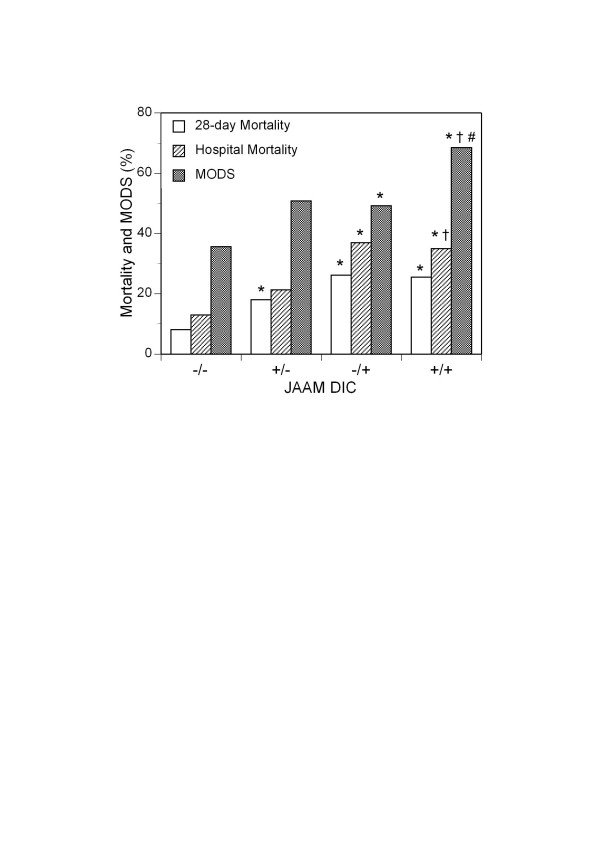
**Twenty-eight-day and hospital mortality rates and prevalence of multiple organ dysfunction syndrome on day 4**. The 28-day and hospital mortality rates and prevalence of multiple organ dysfunction syndrome (MODS) in patients who did or did not exhibit improvement of the Japanese Association for Acute Medicine (JAAM) disseminated intravascular coagulation (DIC) score on day 4: -/-, non-JAAM DIC both on days 1 and 4; +/-, improvement of JAAM DIC on day 4; -/+, proceed to JAAM DIC on day 4; +/+, JAAM DIC on days 1 and 4. Open bars, 28-day mortality; hatched bars, hospital mortality; dark bars, MODS. *P <0.05 versus -/-. ^†^*P *<0.05 versus +/-. ^#^*P *<0.05 versus -/+.

The 28-day and hospital mortality rates also increased in accordance with the increase in the JAAM DIC scores. Furthermore, there was a notable difference in mortality between the patients with scores of 0 to 3 (non-DIC) and those with scores ≥4 (DIC). To predict the 28-day mortality based on the JAAM DIC score on day 1, a receiver operating characteristic curve was constructed. The area under the receiver operating characteristic curve (standard error) and the 95% confidence interval were 0.629 (0.03) and 0.572 to 0.687 (*P *<0.001), respectively. Table 5 shows that the JAAM DIC score on day 1 (the day of diagnosis of severe sepsis) is an independent predictor of 28-day mortality. The Delta JAAM DIC score, defined as the day 1 score minus the day 4 score, was also found to predict 28-day death, which indicates that improvement of DIC is associated with better prognosis of severe sepsis. Figure 1 demonstrates that the time course of JAAM DIC - namely, improvement, deterioration and unchanging scores - significantly affects the prevalence of MODS and the 28-day and hospital mortality rates in patients with severe sepsis. Newly developed and unchanging DIC conditions clearly increased the prevalence of MODS and worsened both mortality rates, which supports the results of the logistic regression analyses.

**Table 5:** Stepwise logistic regression analyses on day of inclusion (day 1) for prediction of 28-day mortality

	Odds ratio	*P *value	95% confidence interval
Age	1.022	0.010	1.005 to 1.040
JAAM DIC score	1.282	<0.001	1.141 to 1.439
Delta JAAM DIC score	0.770	<0.001	0.675 to 0.878
Fibrinogen	0.998	0.033	0.997 to 1.000

DIC, disseminated intravascular coagulation; JAAM, Japanese Association for Acute Medicine. Dependent variables at step 1: age, gender JAAM DIC score, Delta JAAM DIC score (day 1 - day 4), platelet count, prothrombin time ratio, activated partial thromboplastin time, fibrinogen.

### Dynamic changes in platelet counts and DIC diagnosis

In addition to a static assessment, the JAAM DIC scoring system dynamically evaluates changes in platelet counts. A 30 to 50% decrease and a ≥50% decrease in the platelet count within 24 hours add 1 and 3 points to the score, respectively. In the present study, 30 patients exhibited 30 to 50% decreases in the platelet counts and received 1 point, while 32 patients exhibited ≥50% decreases in the platelet counts and received 3 points. Dynamic changes in platelet counts therefore contributed to the diagnosis of DIC in 21.2% (62/292) of the JAAM DIC patients.

### JAAM DIC and ISTH overt DIC in patients with severe sepsis

**Figure 2:**
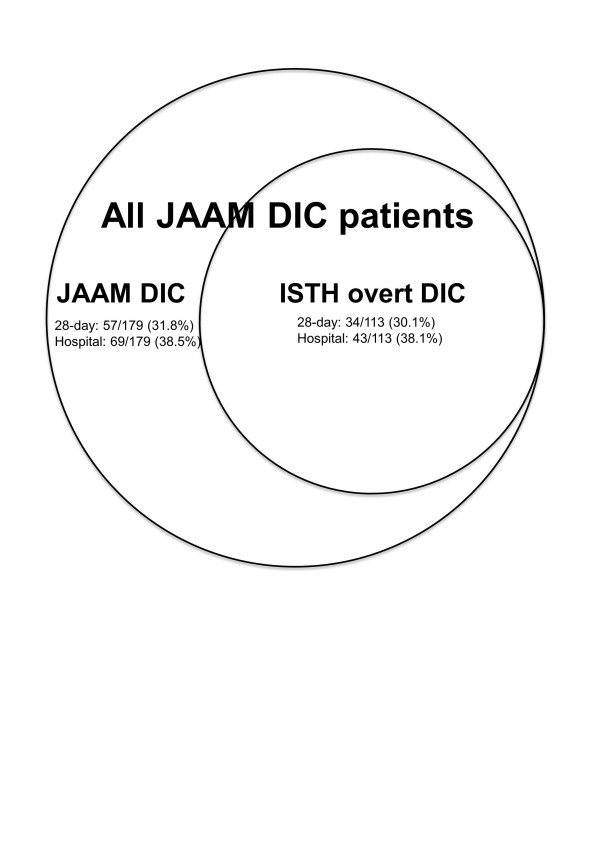
**Relationships between disseminated intravascular coagulation scoring systems**. Japanese Association for Acute Medicine (JAAM) disseminated intravascular coagulation (DIC) and International Society on Haemostasis and Thrombosis (ISTH) overt DIC refer to JAAM DIC patients who met only the JAAM DIC criteria and the JAAM DIC patients who simultaneously met the ISTH overt DIC criteria on day 1, respectively. The numerators in the fractions indicate the patients who died on day 28 (upper) and in hospital (bottom). The ISTH overt DIC scoring system missed dozens of DIC patients on day 1, who would eventually die.

Table 6 shows a comparison between the non-DIC patients, the JAAM DIC patients (those who met the JAAM DIC criteria alone), and the ISTH overt DIC patients (those who simultaneously met both the JAAM and ISTH criteria) on day 1. The JAAM DIC scoring system was able to diagnose and include all of the ISTH overt DIC patients on day 1. Figure 2 represents the relationship between the JAAM DIC and ISTH overt DIC patients. When using the ISTH overt DIC criteria as the gold standard for DIC diagnosis, the sensitivity and specificity of the JAAM DIC scoring system were 100% and 65.0%, respectively. The JAAM DIC patients who simultaneously met the ISTH overt DIC criteria exhibited higher SOFA scores and a higher incidence of MODS. The results in Table 6 and Figure 2 suggest that the ISTH overt DIC criteria missed 57 and 69 DIC patients who will die on the 28th day and in hospital, respectively.

**Table 6:** Comparison between non-DIC, JAAM DIC and ISTH overt DIC on day of inclusion (day 1)

	All JAAM DIC			
	
	Non-DIC (*n *= 332)	JAAM DIC (*n *= 179)	ISTH overt DIC (*n *= 113)	*P *value
Age (years)	69 ± 18	70 ± 15	69 ± 16	0.484
Gender (male/female)	210/122	116/63	65/48	0.432
Septic shock (%)	35.5	51.4	63.7*	<0.001
Positive blood culture (%)	33.9	42.7	60.2**	<0.001
JAAM DIC score	1.9 ± 0.9	5.0 ± 0.9	6.7 ± 1.3***	<0.001
ISTH overt DIC score	1.3 ± 1.2	3.2 ± 0.9	5.7 ± 0.8***	<0.001
SIRS score	3.1 ± 0.9	3.2 ± 0.8	3.4 ± 0.8	0.012
APACHE II score	21.9 ± 7.8	24.6 ± 8.1	26.3 ± 9.0	<0.001
SOFA score	6.7 ± 3.3	9.9 ± 3.8	11.7 ± 3.5***	<0.001
MODS (%)	40.4	60.9	72.6*	<0.001
28-day outcome (death/%)	53/16.0	57/31.8	34/30.1	<0.001
Hospital outcome (death/%)	72/21.7	69/38.5	43/38.1	<0.001

APACHE, Acute Physiology and Chronic Health Evaluation; DIC, disseminated intravascular coagulation; ISTH, International Society on Haemostasis and Thrombosis; JAAM, Japanese Association for Acute Medicine; MODS, multiple organ dysfunction syndrome; SIRS, Systemic inflammatory response syndrome; SOFA, Sequential Organ Failure Assessment. *P *values are between three groups. ISTH overt DIC refers to the patients meeting both the JAAM and the ISTH overt DIC criteria. *P <0.05, **P <0.01, ***P <0.001 versus JAAM DIC.

## Discussion

Diagnostic criteria should meet three conditions: they should be readily available and easy to use, they should exhibit diagnostic accuracy and they should display prognostic value. The JAAM DIC scoring system consists of SIRS criteria, platelet counts and global markers of coagulation and fibrinolysis, is easy to use at the bedside and is commonly available in all hospitals worldwide. The absence of a 100% accurate gold standard for the diagnosis of DIC is a serious limitation in the evaluation of the diagnostic accuracy of DIC diagnostic scoring systems. The validation of the ISTH overt DIC scoring system adopted the opinions of two independent experts in hemostasis and intensive care medicine as the gold standard for the diagnosis of DIC [11]. The diagnostic accuracy of the JAAM DIC scoring system has been evaluated according to two existing systems, the Japanese Ministry of Health and Welfare and the ISTH overt DIC scoring systems, as the gold standard and was confirmed to possess high sensitivity and moderate specificity for DIC diagnosis [8]. Furthermore, the JAAM scoring system is able to diagnose DIC earlier than the two existing systems. The prognostic value of the JAAM scoring system in the critical care setting has also been prospectively confirmed in two previous studies [8,10]. These results were obtained in diverse populations of patients with critical illnesses.

The present study further demonstrated that the JAAM DIC scoring system exhibits good prognostic value in patients with severe sepsis. The patients who met the JAAM DIC criteria on the first day of diagnosis of severe sepsis clearly showed severe systemic inflammation, a higher incidence of MODS, fewer survival days and a higher mortality rate. These changes occurred in parallel with increases in the JAAM DIC scores from 0 to a maximum of 8. The logistic regression analysis revealed a higher 28-day mortality rate in the DIC patients. The SIRS, SOFA and APACHE II scores and the incidence of MODS were in good agreement with our retrospective analysis of the JAAM scoring system in patients with sepsis [23]. However, the mortality rate of the DIC patients observed in the present study was higher than that reported in the previous study due to the greater severity of severe sepsis compared with sepsis. In contrast to these results, Singh and colleagues failed to discriminate between survivors and nonsurvivors among JAAM DIC patients with sepsis [24]. The difference may arise from the lower disease severity (APACHE II score, 14.3 vs. 25.2; SOFA score, 8.3 vs. 10.6) and the extremely higher mortality rate (64.3% vs. 31.2%) in their study subjects compared with that observed in the present study.

The present study showed that in addition to static assessment of the DIC score, dynamic changes in DIC scores are useful for predicting the prognosis of patients with severe sepsis. As presented in Figure 1, the prognoses of patients with severe sepsis worsened more significantly in the patients who proceeded to develop new DIC on day 4 and in those showing continuous DIC from days 1 to 4 than in the non-DIC patients and those recovering from DIC. A logistic regression analysis demonstrated that the Delta JAAM DIC score is an independent predictor of 28-day death among patients with severe sepsis. These results suggest that performing repeated daily scoring, as well as early scoring, is essential in the monitoring of severe sepsis associated with DIC [5,9]. The results also indicate that DIC is a syndrome that should be treated early in addition to managing underlying severe sepsis.

Dynamic DIC scores that take into account temporal dynamic changes in platelet counts and coagulation parameters have recently been proposed [25,26]. The prognostic value of these scores has been assessed in patients with severe sepsis, and the scores have been found to provide useful prognostic information for such patients and to help predict a poor prognosis. Although its prognostic value has not been confirmed, the reduction rate of platelets contributed to DIC diagnosis in 21% of the patients with JAAM DIC in this study. It is therefore necessary to be aware that, due to the wide distribution of normal counts, the platelet counts may remain within the normal range during the early stage of DIC. In such situations, a continuous drop and/or the rate of decrease in the platelet count over consecutive measurements are more important for diagnosing DIC than are the absolute values [8].

In the present study, the JAAM DIC scoring system was able to diagnose all of the ISTH overt DIC patients on the day of diagnosis of severe sepsis. The patients who met both the JAAM and the ISTH overt DIC criteria exhibited higher SOFA scores and more complications with MODS. The ISTH overt DIC scoring system missed 126 (57 and 69) severe sepsis patients who will die on the 28th day or in hospital. Similarly, the ISTH overt DIC scoring system was not able to detect 79% of nonsurvivors with non-overt DIC [27]. Taken together, these results indicate that the JAAM DIC scoring system may be more useful than the ISTH overt DIC scoring system for selecting DIC patients with a poor prognosis as well as those requiring treatment among the population of severe sepsis patients, which coincides with our former results [8].

The present study primary evaluated the prognostic value of the JAAM DIC scoring system, while the diagnostic accuracy was not assessed due to the lack of a gold standard for the DIC diagnosis. However, we confirmed the high sensitivity and moderate specificity of the JAAM scoring system using the ISTH overt DIC criteria as the gold standard. The ISTH overt DIC criteria may be too strict to select the patients who will eventually die and may require the assistance of a non-overt DIC scoring system [9]. We believe that these results indirectly indicate the DIC diagnostic accuracy of the JAAM DIC diagnostic algorithm in the early phase of severe sepsis in a critical care setting.

This study does have limitations. The methods used for the measurement of the FDP were not unified between the participating hospitals in the present study. Differences in the ranges of the FDP levels between the hospitals were also not considered. These limitations might have introduced a bias in the results of the present study.

## Conclusions

In the critical care setting, the JAAM DIC scoring system exhibited superior prognostic value in predicting MODS and poor prognoses in patients with severe sepsis. The JAAM DIC scoring system is able to select more patients with DIC who require treatment and are near death than the ISTH overt DIC scoring system. In addition to conducting a static assessment of the DIC score, scoring should proceed daily in order to evaluate the severity and development of DIC and to predict the patient's prognosis. Dynamic scoring of platelet counts in the JAAM DIC scoring system in part contributes to the sensitivity of this system in the diagnosis of DIC.

## Key messages

• The JAAM DIC scoring system exhibits good prognostic value in predicting MODS and poor prognosis in patients with severe sepsis.

• The JAAM DIC scoring system can detect more patients requiring DIC treatment in patients with severe sepsis.

• Conducting repeated daily JAAM scoring increases the ability to predict the prognosis of the patients with severe sepsis.

## Abbreviations

APACHE II: Acute Physiology and Chronic Health Evaluation II; DIC: disseminated intravascular coagulation; FDP: fibrin/fibrinogen degradation product; ISTH: International Society on Haemostasis and Thrombosis; JAAM: Japanese Association for Acute Medicine; MODS: multiple organ dysfunction syndrome; SIRS: systemic inflammatory response syndrome; SOFA: Sequential Organ Failure Assessment.

## Authors' contributions

SG, DS, HO, SF, TA, HI, JK, SK, YM, SS, KS, YS, NT, KT, RT, YY, NY and NA participated in the study design and data interpretation, and helped to draft the manuscript. SG, HO, SF, TM, HI, JK, SK, SS, KS, NT, KT, RT, YY and NY collected data. SG wrote the manuscript. DS performed the statistical analyses. All authors read and approved the final version of the manuscript.

## Competing interests

The authors declare that they have no competing interests.
